# Financial capability and functional financial literacy in young adults with developmental language disorder

**DOI:** 10.1177/2396941518794500

**Published:** 2018-08-15

**Authors:** Maxine Winstanley, Kevin Durkin, Roger T Webb, Gina Conti-Ramsden

**Affiliations:** The University of Manchester and Manchester Academic Health Science Centre, UK; University of Strathclyde, UK; The University of Manchester and Manchester Academic Health Science Centre, UK

**Keywords:** Financial capability, financial literacy, developmental language disorders, young adulthood

## Abstract

**Background:**

Financial capability is an essential feature of the organisation of one’s personal life and engagement with society. Very little is known of how adequately individuals with developmental language disorder handle financial matters. It is known that language difficulties place them at a disadvantage in many aspects of their development and during their transition into adulthood, leading to the possibility that financial issues may prove burdensome for them. This study examines the financial capability and functional financial literacy of young adults with developmental language disorder and compares them to those of age matched peers. We tested the expectation that those with developmental language disorder would find financial management more challenging than would their peers, and that they would need to seek greater support from family members or other people.

**Methods:**

Participants completed a detailed individual interview, which included items drawn from the British Household Panel Survey and additional measures of financial capability, functional financial literacy and of perceived support. Nonverbal IQ, language, reading and numeracy measures were also collected.

**Results:**

Compared to typically developing age matched peers, young people with developmental language disorder report less extensive engagement with financial products and lower competence in functional financial literacy. A considerably higher proportion of those with developmental language disorder (48% vs. 16% of age matched peers) report that they draw on support, primarily from parents, in various financial tasks, including paying bills, choosing financial products, and taking loans from family or friends.

**Conclusions:**

This is the first study to consider the financial capability skills and functional financial literacy of young adults with developmental language disorder. We provide novel evidence that some young adults with developmental language disorder lack functional financial skills and require support to successfully manage their finances. This has policy implications that relate not only to engaging affected individuals in discussions about financial management but also to wider familial support.

## Introduction

Financial capability entails conceptual and terminological understanding, as well as the management of one’s own monetary affairs (Atkinson, McKay, Kempson, & Collard, 2006; Taylor, Jenkins, & Saker, 2011). Ever more so in the context of economic crises, prolonged periods of austerity and insecure employment, contemporary young people face considerable hazards in handling financial matters. Increasing the level of financial capability, particularly amongst the most vulnerable in society, has become a target for national governments (Allmark & Machaczek, 2015; Xiao, Chen, & Sun, 2015). Evidence indicates that those with greater financial competence tend to enjoy greater psychological wellbeing (Melhuish, Belsky, & Malin, 2008; Taylor et al., 2011), more stable financial behaviour (Hilgert, Hogarth, & Beverley, 2003; Lusardi & Mitchell, 2007) and achieve more favourable economic outcomes (Lusardi & Mitchell, 2014).

## Developmental language disorder

Developmental language disorder (DLD) refers to a difficulty with language that is not accounted for by physical, cognitive and/or neurological causes (Bishop, Snowling, Thompson, Greenhalgh, & CATALISE-2 CONSORTIUM, 2017; Durkin & Conti-Ramsden, 2010). It affects approximately 7% of children on school entry (Tomblin, Records, & Zhang, 1997). DLD is not exclusive to childhood but can continue into adolescence and adulthood (Clegg, Hollis, Mawhood, & Rutter, 2005; St Clair, Pickles, Durkin, & Conti-Ramsden, 2011; Yew & O’Kearney, 2013). Except for severe cases, adolescents with DLD are less easy to identify, often relying on facilitative strategies to mask their difficulties (Durkin & Conti-Ramsden, 2010). Thus, adolescents with DLD, despite precarious language skills, are often able to get by in familiar everyday interactions without their difficulties becoming readily apparent to others (Durkin & Conti-Ramsden, 2010).

Despite the somewhat hidden nature of this disability, longitudinal studies demonstrate that adolescents and young adults with DLD demonstrate poorer outcomes, when compared with their peers, in multiple domains that go beyond language understanding and use (Conti-Ramsden & Durkin, 2008, 2012; Johnson, Beitchman, & Brownlie, 2010). These broader disadvantages are likely also to have implications for how well young people with DLD are equipped to deal with financial matters. For example, the terminological and conceptual requirements of this domain may pose difficulties for those with poorer language, reading and numerical skills. Language abilities impact on many aspects of educational progress, including literacy (Botting, Crutchley, & Conti-Ramsden, 1998; Snowling, Adams, Bishop, & Stothard, 2001) and numeracy (Cowan, Donlan, Newton, & Lloyd, 2005; Durkin, Mok, & Conti-Ramsden, 2013, 2015). Both reading, and numeracy are drawn upon substantially in tasks such as processing literature about financial products, making purchases, form filling, and communications with relevant bodies (Grohmann, Kouwenberg, & Menkhoff, 2015; LeFevre et al., 2010). Hence, there are grounds to expect talking, reading and making decisions and calculations about money to be challenging for those with DLD.

Similarly, in general, adolescents and young adults with DLD tend to lag behind their typical peers in terms of attaining independence in everyday life skills (Conti-Ramsden & Durkin, 2008). They tend to be less self-efficacious in various domains (Botting, Durkin, Toseeb, Pickles, & Conti-Ramsden, 2016), they fare less well on entry to the job market (Conti-Ramsden & Durkin, 2012) and are less socially confident (Durkin, Toseeb, Pickles, Botting, & Conti-Ramsden, 2017). Self-efficacy is associated with financial capability (Xiao et al., 2015) and social confidence is associated with mastery of everyday personal and occupational tasks (Durkin et al., 2017; Smith & Betz, 2000). These considerations suggest that young people with DLD will be less capable of meeting the diverse demands of financial management and may well need greater support in this domain than do their typically developing peers.

Emerging evidence suggests that providing early targeted invention to certain groups may aid in ameliorating later adverse outcomes. Initiatives including Head Start, aimed at reducing the educational gap for disadvantaged families in the USA, report favourable long-term outcomes in domains considered distal to the original intervention. For example, longitudinal studies have reported less obesity, depression (Currie & Neidell, 2007) and offending (Carneiro & Ginja, 2014) from cohorts who participated compared to disadvantaged families that did not. In the same vein, Winstanley, Webb, and Conti-Ramsden (2018) found a high prevalence of unidentified DLD in a young offender population, although in young adults with identified DLD who had received early targeted intervention in language units they found there were less adversarial contacts with their local police service. Thus, it is possible that early professional intervention may confer an environment whereby optimum outcomes are realised.

Evidence on financial capability in young people with DLD is limited. Conti-Ramsden and Durkin (2008) found that, for adolescents with DLD, parents reported that 74% could manage money, while 94% of parents of typically developing adolescents perceived their offspring as capable in this domain. Note that, at this age (16 years), money management is likely to be more elementary (e.g. dealing with pocket money, income from part-time work) than in early adulthood, a time when young people are dealing with a wider range of self-organisational matters and more factors external to the parental home. Furthermore, there was some discrepancy between parental reports and self-reports. Adolescents with DLD themselves were more confident, with 86% reporting that they were able to manage money (typically developing adolescents, at 98%, were only marginally more likely to see themselves as competent in this respect than their parents estimated them to be). These findings suggest that, at least in adolescence, some young people with DLD may have been unaware of their own limitations concerning money management and/or that they underestimated the contributions of any parental support that they may have been obtaining. Conti-Ramsden and Durkin, however, had only one item concerning money and there is a need for more wide-ranging measures, which the present study provides. This investigation examines financial capability during the more testing period of early adulthood and develops a reliable measure of functional financial literacy that examines young adults’ abilities to deal with everyday monetary transactions. We expected that financial capability of young adults with DLD would be limited when compared to that of AMPs and, in turn, that functional financial literacy would be particularly poor in individuals with DLD. We also posit that young people with DLD would report obtaining more support with financial management than their AMPs. Finally, we hypothesised that language, reading and numeracy would all be significantly associated with functional financial literacy in young adults with DLD.

## Method

### Ethics

The study reported here received ethical approval from The University of Manchester. All participants provided informed written consent.

### Participants

*Young adults with DLD:* The reported study focuses on young adults at age 24 years, all of whom had a history of identified DLD (referred to henceforth as ‘young adults with DLD’ for ease of reading). The participants were originally part of the longitudinal Manchester Language Study (MLS), which examined an initial cohort of 242 children (Conti-Ramsden, Crutchley, & Botting, 1997). These children represented a random sample of 50% of all 7-year olds attending 118 language units from across England for at least half of the school week.

Participants were contacted at ages 8 (n = 232), 11 (n = 200), 14 (n = 113), 16 (n = 139), and 24 (n = 84). Funding constraints contributed to the attrition at these follow up stages. The current sample, 35% of the original cohort, consisted of 56 (67%) males and 28 (33%) females, ranging in age between 23.4 years and 25.9 years (*M* = 24.4; *SD* = 0.7 years). To examine potential attrition bias, we compared the receptive language, expressive language, nonverbal IQ and gender distribution of individuals with a DLD who continued to participate at 24 years and those who did not. There were no significant differences in receptive language (*t*(240) = −1.13, *p* = .261), expressive language (*t*(229) = −0.45, *p* = .654), and nonverbal IQ (*t*(231) = −0.60, *p* = .547) standard scores at age 7 between those who participated at age 24 and those who did not. At age 24 years, the gender distribution in the DLD group (67% male; 33% female) was not significantly different from that of the comparison group (56% male; 44% female, see below), χ^2^(1, *N* = 172) = 2.18, *p* = .140.

*Aged-matched peers (AMPs):* The comparison group consisted of 88 AMPs, 49 (56%) of whom were males and 39 (44%) of whom were females, ranging in age between 22.3 years and 26.0 years (*M* = 24.1; *SD* = 0.9 years). The comparison group had no history of receiving speech or language therapy or of special educational needs provision (as ascertained by teacher report). Sixty-six of these young adults were recruited at age 16 years and 22 young adults were recruited for the age 24 wave of the MLS. The age 16 participants were recruited from the same schools as the participants with DLD as well as additional targeted mainstream schools. For the age 24 wave, areas with specific sociodemographic profiles were selected for sampling and recruiting comparison peers so that their backgrounds would be similar to the participants with DLD. Thus, the 22 young adults recruited matched the original sample in terms of age and socioeconomic status as measured by personal income. All participants had remained in school until the end of compulsory education (in the UK, at 16 years on average). The DLD and the AMP groups did not differ on household income at age 16 years (χ^2^(10, *N* = 145) = 9.32, *p* = .501) or on personal income at age 24 years (χ^2^(5, *N* = 131) = 7.38, *p* = .194).

*Psycholinguistic profiles of participant groups:* Comparisons of mean standard scores for participants with DLD vs. AMPs, including standard deviations, are presented in Table 1. All scores for the AMPs were within the expected range. The mean language scores for the young adults with DLD was more than 1 standard deviation below the mean (<.85). Mean nonverbal IQ scores were within the expected range and close to the population average. The participants with DLD, however, had significantly lower nonverbal IQ scores than their peers. Evidence suggests that the nonverbal abilities of individuals with DLD may decline in adolescence (Botting, 2005; Leonard, 2014).

**Table 1.** Psycholinguistic profiles of participant groups.

	Group					
	DLD Range (N = 84)	AMP Range (N = 88)	*t*	*df*	Mean difference [95% CI]	Cohen’s *d*
Nonverbal IQ (SS)	98.8 (15.8) 55–131	111.9 (10.3) 79–129	6.4***	167	13.1 [9.1, 17.2]	1.1
*Language (SS)* Expressive language (SS) Receptive language (SS)	82.4 (17.3) 40–115 81.6 (19.0) 55–120 83.5 (18.6) 55–115	105.9 (9.3) 56–124 105.6 (8.9) 55–120 106.2 (8.9) 65–115	11.0*** 9.8*** 10.1***	167 167 168	23.6 [19.3, 27.8] 24.1 [19.3, 28.8] 22.7 [18.3, 27.1]	1.7 1.5 1.6
*Reading (RS)* Reading accuracy (RS) Reading comp (RS)	34.7 (6.1) 14–45 43.7 (7.6) 19–55 25.4 (6.0) 8–38	42.3 (2.8) 31–47 52.2 (3.3) 34–55 31.9 (3.1) 23–38	10.5*** 9.5*** 8.8***	167 168 167	7.6 [6.2, 9.0] 8.5 [6.7, 10.3] 6.5 [5.0, 7.9]	1.6 1.4 1.4
Numeracy (SS)	75.2 (13.7)	90.4 (12.5)	7.5***	168	15.2 [11.2, 19.2]	1.2

DLD: Developmental Language Disorder; AMP: Aged-matched Peers; SS: Standard Scores; RS: Raw Scores.

*** *p* ≤ .001.

## Materials and measures

### Psycholinguistic measures of nonverbal, language, reading and arithmetic skills

#### Nonverbal IQ

The Wechsler Abbreviated Scale of Intelligence (WASI, Wechsler, 1999) Performance subscale was administered as a measure of nonverbal IQ and standard scores were calculated. This test has norms for individuals aged 6–89 years. The reliability of the Performance IQ scale for the age range 20–24 years is .94. Validity studies of the WASI reported in the manual provide evidence that the test is a valid quick screening measure of intellectual functioning.

#### Language

To assess language ability, the Clinical Evaluation of Language Fundamentals (CELF-4^uk^) (Semel, Wiig, & Secord*,* 2006) was utilised. The CELF-4 is a standardised assessment and is normed up to age 21 years 11 months. Despite the slightly older age of this current cohort compared to the normed age of the test, no participant reached ceiling level. This, coupled with the lack of standardised language assessments available for adults, meant the CELF-4 was deemed the most suitable instrument. Two sub-tests of the CELF-4 were utilised, consisting of word classes receptive language measure (WCR) which requires the participant to listen to a list of four words and decide which two are related. This sub-test relies on the ability to comprehend associations among words and is concerned with the structural aspect of language. Formulating sentences expressive language subtest (FS) requires the participant to formulate a sentence, including a given word, based on a picture shown. This measures the ability to articulate in a coherent logical order illustrating both vocabulary use and sentence structure. For the age range 17.0–21.11 years, the reliability of the WCR subtest was .88 and for the FS subtest it was .82. Clinical validation studies of the CELF-4 reported in the manual indicate that the test is sensitive to language impairment in children, adolescents and young adults. The overall measure of language used for analyses was based on the mean of the two CELF-4 sub-tests.

#### Reading

Basic Reading (tapping reading accuracy) and the Reading Comprehension subtests of the Wechsler Objective Reading Dimensions (WORD, Wechsler, 1993) were used to obtain an overall reading score. This score was calculated as the mean of these two subtests. As this test only provides normative data up to 16.11 years, raw scores were used for analyses purposes. The WORD manual details good reliability (basic reading: .91; reading comprehension: .86) and validity (basic reading: .80; reading comprehension: .81).

#### Numeracy

Numeracy was assessed using the arithmetic subtest of the Wide Range Achievement Test – Third edition (WRAT-3; Wilkinson, 1993). This test can be used with people aged 5–75 years. The WRAT-3 has been found to have good reliability (.92 to .93) and validity (.83 to .87).

### Measure of financial capability

We examined three of the strongest dimensions comprising financial capability discussed in the literature: ‘managing money’, ‘planning ahead’ and ‘making choices’ (Atkinson et al., 2006). We operationalised ‘making choices’ with a measure of engagement with financial products (referred to as ‘financial products’ henceforth). We utilised, and supplemented, questions from the British Household Panel Survey (University of Essex; BHPS Waves 1–18, 1991–2009), to capture these three different dimensions of financial capability.

#### Managing money

Managing money was measured with three questions. We utilised a survey question from the BHPS that has previously been adopted in empirical studies (Atkinson et al., 2006; Taylor et al, 2011) and added two bespoke questions. Following the BPHS we asked: ‘How well would you say you yourself are managing financially these days?’ Responses were provided on a scale from 1 (‘finding it very difficult’) through to 5 (‘managing comfortably’). We then asked two further questions: ‘Do you know your monthly expenditure?’ (taking the values 0 for ‘no’ and 1 for ‘yes’), and ‘Do you pay regular bills on time?’ (values ranging from 1 for ‘never’ through to 5 for ‘always’).

#### Planning ahead

To address the dimension of planning ahead, a further question from the BHPS was utilised, i.e. ‘Do you save any amount of your income?’, the values 0 for ‘no’ and 1 for ‘yes’ were ascribed to this question. Additionally, participants were asked ‘How well do you plan your spending’ (taking values from 1 ‘not at all’, to 5 ‘very well’).

#### Financial products

We asked participants about their engagement with nine financial products: mortgages, current accounts, savings accounts (including overdrafts, credit cards, store cards, student loans, finance deals – e.g. for large purchases such as a car or a sofa, and loans – not including student loans). Participants received 1 point for each of the financial products they possessed, yielding a maximum possible score of 9. In addition, we examined how participants accessed financial products by asking: ‘How do you access your financial products most often?’. The options included online banking, telephone banking, face-to-face services, ATM machines (referred to colloquially as ‘hole in the wall’), or other services.

### Measures of functional financial literacy

We were also interested in examining everyday functional aspects of managing money in early adulthood for individuals with DLD, i.e. functional financial literacy. This was measured by three bespoke questions designed to consider an individual’s ability to manage everyday monetary transactions and decipher financial data in real time. Participants were asked: ‘Can you easily add up the cost of several items before you pay for them?’, ‘Can you work out in advance what change you might get?’, and ‘Can you easily work out which brand is the best value for money?’. Each question was scored on a scale from 1 for ‘not often’, 2 for ‘sometimes’, and 3 for ‘most of time’; the higher the score, the better the young person’s functional financial literacy skills. Results from Cronbach’s alpha, indicated reliability was very good, α = 0.80, for this measure.

### Measures of financial support

Participants were asked a series of questions pertaining to support they obtained with their finances. First, they were asked if they obtained any regular support and, if so, from whom. Four potential sources were offered: parent, partner, friend or other. Participants who reported that they did receive support were then asked to indicate, from a list, the particular type of support. The list included: help with paying bills, choosing financial products, applying for financial products, managing money and managing debt. Respondents were also asked if they had ever sought financial help from friends and family in the form of a loan.

### Statistical analysis

The data were analysed using SPSS Version 20. Comparisons were based on two-sample *t*-tests and for categorical variables, Chi-squares (see Tables 2 and 3). Associations were examined using Pearson correlation analyses. Univariate and multivariable linear regression models were fitted to examine predictors of functional financial literacy. Group membership (DLD coded as 0 and AMP coded as 1) was included as an independent (predictor) variable. When conducting sensitivity analysis, we applied a Bonferroni correction for the different financial capability measures, yielding a corrected two-tailed significance level of *p* = .005.

**Table 2.** Mean scores and group differences for financial capability.

Financial capability	DLD Mean (SD) Range/% yes/no *n*	AMP Mean (SD) Range/% yes/no *n*	Test statistic	*p* value	Mean difference [95% CI]	Cohen’s *d*
*Managing money* How well would you say you are managing financially these days?	4.0 (.8) 2–5 *n* = 79	3.9 (.8) 2–5 *n = *87	*t*(164) = .6	.54	.08 [−.17, .32]	
Do you know your monthly expenditure?	Yes = 80% No = 20% *n* = 80	Yes = 92% No = 8% *n* = 87	χ^2^(1, *N* = 167) = 5.0	.03		
Do you pay regular bills on time?	4.9 (.4) 3–5 *n* = 60	4.8 (.6) 2–5 *n* = 82	*t*(140) = 1.4	.18	.12 [−.05, .28]	
*Planning ahead* Do you save any amount of your income?	Yes = 57% No = 43% *n* = 80	Yes = 56% No = 44% *n* = 87	χ^2^(1, *N* = 167) = .02	.88		
How well do you plan your spending?	3.6 (1.1) 1–5 *n* = 80	3.4 (1.1) 1–5 *n = *87	*t*(165) = 1.5	.14	.26 [−.08, .60]	
*Financial products* Number of products	2.9 (1.3) 1–6 *n* = 80	4.0 (1.5) 1–8 *n* = 85	*t*(163) = −5.0	<.001	−1.09 [−1.51, −.65]	0.78
How products were accessed: Online banking Telephone banking Face-to-face services ATM machines Other services	33% 2% 10% 49% 6% *n* = 82	68% 2% 6% 23% 1% *n* = 87	χ^2^(1, *N* = 169) = 20.6 χ^2^(1, *N* = 169) = .95 χ^2^(1, *N* = 169) = 12.3	<.001 .39 <.001		

DLD: Developmental Language Disorder; AMP: Aged-matched Peers.

Note: Chi-square analyses for ‘how products were accessed’ were only undertaken when the expected frequencies were >5.

**Table 3.** Frequency of support obtained by group.

Measure	DLD		AMP		Chi-square	*p* value
	*N*	Freq	*N*	Freq		
Paying bills	80	12	87	1	11.1	.001
Choosing financial products	80	22	87	4	16.6	<.001
Applying for financial products	80	14	87	1	13.6	<.001
Managing money	80	10	87	1	8.4	.003
Loans from family and friends	80	24	87	10	8.8	.003

DLD: Developmental Language Disorder; AMP: Aged-matched Peers.

## Results

### Financial capability

Results of the three aspects of financial capability are presented in Table 2. There were significant group differences (with medium effect sizes) for financial products, but, in general, not for managing money and planning ahead domains. The one exception was ‘Do you know your monthly expenditure?’. A lower proportion of individuals with DLD (80% vs. 92% for AMPs), responded positively to knowing their monthly expenditure. Applying the Bonferroni correction, when conducting sensitivity analysis, meant this variable was no longer statistically significant.

### Functional financial literacy

An independent-samples *t*-test comparing the mean functional financial literacy scores of the two groups found a significant difference between the means of the two groups *t*(165) = −8.45, mean difference = −1.88 [95% CI = −2.32, −1.44], *p* ≤ .001, with a large effect size (Cohens’ *d = *1.46). The mean of the DLD group was significantly lower (*m* = 6.91, *SD* = 1.92) than the mean of the AMP group (*m* = 8.79, *SD* = .749).

### Support with finances

Almost half, 48% (38/80) of the DLD group reported obtaining support, contrasting with 16% (14/87) of AMPs. This difference was significant, χ^2^ (1, *N = *167) = 19.17, *p* ≤ .001. The majority of this support, in both groups, came from parents. Within the DLD group 43% (36/84) reported receiving support from their parents whilst only 14% (12/88) of AMPs reported receiving this support, χ^2^ (1, *N = *167) = 18.24, *p* ≤ .001. Table 3 details the types of support regarding finances obtained by participants. For each type of support, there were significant group differences.

### Examination of associations

We examined the relationships between the psycholinguistic characteristics of the participants and functional financial literacy. Because financial literacy showed large between-groups difference, we report the correlations for each group separately. The findings are presented in Table 4. In terms of functional financial literacy, the only association in the AMP group was with numeracy (*rs* = .26, *p* = .02), this was also evident in the DLD group (*rs* = .55, *p* ≤ .001), but additionally, functional financial literacy was associated with language, literacy and nonverbal IQ.

**Table 4.** Associations between functional financial literacy and other abilities.

	1	2	3	4	5
1. Functional financial literacy	1				
2. Nonverbal IQ	DLD: 0.54*** AMP: 0.16	1			
3. Core language	DLD: 0.32*** AMP: −0.05	DLD: 0.53*** AMP: 0.32**	1		
4. Overall reading	DLD: 0.29** AMP: 0.03	DLD: 0.44*** AMP: 0.40***	DLD: 0.79*** AMP: 0.50***	1	
5. Numeracy	DLD: 0.55*** AMP: 0.26*	DLD: 0.68*** AMP: 0.55***	DLD: 0.65*** AMP: 0.26*	DLD: 0.59*** AMP: 0.41***	

DLD: developmental language disorder; AMP: age matched peers.

* p < .05.

** p < .01.

*** p < .001.

### Predictors of functional financial literacy

A multivariable linear regression was performed with functional financial literacy as the outcome. The predictors were the psycholinguistic variables and group status (DLD coded as 0 and AMP coded as 1). Multicollinearity tests indicated that all the variance inflation factors (VIFs) were all below 4 and tolerance was never less than 0.2 for any of the covariates. An analysis of standardised residuals identified one outlier, with casewise diagnostics revealing that this outlier was more than 3 standard deviations below the mean. Removal of the outlier had no effect on the pattern or results or significance levels; therefore, the data point was retained. Table 5 presents the results. The adjusted *R*^2^ values showed that together the predictors accounted for 44% of the variance of functional financial literacy (*F* (5,164) = 26.70, *p* ≤ .001; adj. *R*^2 ^= .44). Three variables emerged as significant predictors: nonverbal IQ, numeracy and group status. Comparisons of the standardised regression coefficients suggested that the effect of group status accounted for the greatest proportion of variance in the model, with functional financial literacy scores decreasing by almost a third with DLD group membership.

**Table 5.** Regression analysis for variables predicting functional financial literacy skills in young adults at age 24.

Predictors	Unstandardised coefficients			Standardised coefficients	*p* value
	B	Std. error	95% CI	Beta	
(Constant)	3.222	1.035			.002
Nonverbal IQ	.026	.010	.006, .046	.221	.01
Core language	−.005	.011	−.027, .017	−.053	.65
Reading	.007	.032	−.055, .069	.025	.82
Numeracy	.032	.010	.012, .052	.292	.002
Group	−1.086	.263	1.605, −.568	−.324	<.001

To investigate these group differences further, separate regression analyses were carried out for the two groups separately. Results are shown in Table 6. For the DLD group, the regression was significant (*F* (4,78) = 9.763, *p* ≤ .001; adj. *R*^2 ^= .31), accounting for 31% of the variance with numeracy as the only significant predictor. The regression equation for the AMP group was non-significant (*F* (4,85) = 1.755, *p* = .146; adj. *R*^2 ^= .03). We ran a second sensitivity analysis, omitting the participants with core language scores falling outside of the range expected for their group status. This revealed the same set of results; for the DLD group the regression was significant (*F*(4,31) = 6.021, *p* ≤ .001; adj R = .33), but it was not significant for the AMP group (*F*(4,78) = 1.763, *p* = .145; adj R = .04). See Appendix 1, Table 7 for the regression table.

**Table 6.** Regression analysis for variables predicting functional financial literacy in young adults at age 24 by group status.

Predictors	Unstandardised coefficients			Standardised coefficients	*p* value
	B	Std. error	95% CI	Beta	
*DLD group*					
(Constant)	.290	1.286			.82
Nonverbal IQ	.031	.016	−.001, .064	.257	.054
Core language	−.020	.019	−.057, .017	−.182	.28
Reading	.014	.048	−.082, .111	.047	.77
Numeracy	.062	.020	.023, .102	.463	.002
*AMP group*					
(Constant)	8.184	1.311			<.001
Nonverbal IQ	.004	.010	−.015, .023	.055	.68
Core Language	−.005	.101	−.025, .015	−.058	.65
Reading	−.020	.035	−.090, .049	−.077	.56
Numeracy	.017	.008	.001, .032	.278	.04

DLD: developmental language disorder; AMP: age matched peers.

## Discussion

This is the first study to consider the financial capability skills and functional financial literacy of young adults with DLD. The findings reveal that the consequences of this disability extend to important practical domains of early adult life. Compared to typically developing AMPs, young people with DLD report less extensive engagement with financial products and lower competence in functional financial literacy. A considerably higher proportion of those with DLD (48% vs. 16% of AMPs) report that they draw on support, primarily from parents, in various financial tasks, including paying bills, choosing financial products, and taking loans from family or friends. Extending earlier evidence of a lag in achieving personal independence during adolescence (Conti-Ramsden & Durkin, 2008), the present study shows that managing everyday financial affairs poses challenges for those with DLD and leaves many in need of continuing parental support.

We did not find the expected difference between DLD and AMP groups in respect of managing money and planning ahead. On first glance, this may appear encouraging, with mean scores (Table 2) indicating that both groups regarded themselves as being reasonably competent at managing money and planning ahead financially. However, this finding should be interpreted cautiously for several reasons. First, approximately half of the participants with DLD also acknowledged that they needed parental support; hence, for them, ‘managing money and planning ahead’ in this context may be socially mediated rather than fully independent activities. Second, and related to the first point, young people participating in this study were individuals with identified DLD who had received early targeted intervention in language units. Such early professional intervention with children and their families may confer an environment that fosters parental support and understanding of individuals’ long-term needs. In this study, therefore, we may be observing optimal outcomes in relation to managing finances (see also Winstanley et al., 2018). Third, the extent and adequacy of managing money and forward planning in either group were not tested, and many young people with or without language difficulties may, for example, plan only for the relatively short-term, especially if they have not received targeted financial education (Xiao & O’Neill, 2016). In this sense, the item regarding knowing monthly expenditure may be an indicator of potential areas of difficulty when planning beyond the short-term. Further evidence on emerging financial performance is needed before we can be confident that those with DLD are as able as their peers in managing money and planning ahead financially.

The findings concerning modes of access to financial products also suggest more limited strategies for money management in those with DLD. They were markedly less likely than their typical peers to use online banking facilities and were more likely to use ATMs. The information technology demands of online access present barriers to people with disabilities (Dobransky & Hargittai, 2006). During mid-adolescence, young people with DLD are less likely than their peers to use the Internet to make purchases (Durkin et al., 2009). On the other hand, those with DLD seem to favour the relatively direct access to cash provided by ATMs. Cash machines are designed to be very user-friendly and most aspects of transactions therein can be completed with simple ‘press option’ actions. Although this is a very widely used and convenient banking mechanism, a possible disadvantage of high dependence upon them is losing track of spending. Alternatively, for some individuals, using cash may provide a more concrete (tangible and visual) means of monitoring their own spending.

Relationships among functional financial literacy and other abilities revealed differences between the groups. In the DLD group, there was a significant association with reading, language and nonverbal IQ. In contrast, for the AMPS, the only association found for functional financial literacy was a weak association with numeracy. In multivariable regression analyses, only in the DLD group did the model provide a good fit to the data, with the explanatory variables accounting for 31% of the variance.

Not surprisingly, functional financial literacy was associated with numeracy in both groups. There is extensive evidence of poorer performance in numeracy and mathematics in children and adolescents with DLD (Cowan et al., 2005; Durkin et al., 2013, 2015; Young et al., 2002). The present findings indicate that the consequences of this relationship extend beyond academic performance, and place young adults with DLD at a disadvantage in managing their finances. Our measure pertaining to functional financial literacy consisted of a series of questions. Future research could consider practical exercises to measure functional financial literacy such as, asking participants to calculate the price of multiple goods or the price when discounted by a certain percentage.

Proficiency in mathematics relies on the understanding of technical domain-specific vocabulary (Lyytinen, Ahonen, & Rasanen, 1994), the ability to decipher complex written problems (Woodward & Peters, 1983) and the understanding that a range of mathematical words can be used interchangeably (Purpura & Ganley, 2014), all of which are linguistic tasks. The hierarchical nature of mathematical knowledge dictates that early difficulties are precursors to more marked difficulties over time (Aunola, Leskinen, Lerkkamem, & Nurmi, 2004). We found an association between numeracy and language across the linguistic range. The statistical correlations (Table 4) suggest that, although this is present irrespective of ability, the association appears to be stronger in the lower range of abilities. Our study adds to previous research and provides evidence that the association of language and numeracy does not attenuate over time among young adults with a history of DLD. Additionally, we provide evidence that that these difficulties manifest in ways that affect subsequent functioning in adult life.

## Conclusions

This study provides unique information pertaining to the financial capability and functional financial literacy skills of young adults with DLD. Young adults with DLD are not excluded from the financial world, but it is (another) aspect of the human environment that can present special challenges to them. The study augments earlier findings that language ability supports the acquirement of arithmetic skills, and this association is much stronger in the DLD group. We provide novel evidence that some young adults with DLD lack functional financial skills and require assistance to be able to manage their finances. This has policy implications that relate not only to engaging those at risk in discussions about financial management but also to wider familial support.

## Appendix 1

**Table 7.** Regression analysis for variables predicting functional financial literacy in young adults at age 24 by group status (sensitivity analysis).

Predictors	Unstandardised coefficients			Standardised coefficients	*p* value
	B	Std. error	95% CI	Beta	
*DLD group*					
(Constant)	−.888	2.249			.70
Nonverbal IQ	.057	.024	.010, .105	.396	.02
Core Language	−.025	.034	−.093, .043	−.126	.46
Reading	−.006	.057	−.122, .110	−.017	.92
Numeracy	.058	.029	.000, .117	.343	.052
*AMP group*					
(Constant)	9.332	1.398			<.001
Nonverbal IQ	−.001	.010	−.022, .020	−.014	.92
Core Language	−.017	.015	−.046, .012	−.163	.25
Reading	−.005	.036	−.077, .067	−.019	.89
Numeracy	.018	.008	.002, .034	.305	.02
